# Ecophysiology of four co-occurring lycophyte species: an investigation of functional convergence

**DOI:** 10.1093/aobpla/plv137

**Published:** 2015-11-24

**Authors:** Jacqlynn Zier, Bryce Belanger, Genevieve Trahan, James E. Watkins

**Affiliations:** Department of Biology, Colgate University, Hamilton, NY 13346, USA

**Keywords:** Clubmoss, ecophysiology, functional traits, Lycopodiaceae, nitrogen, photosynthesis

## Abstract

Lycophytes are the most early divergent extant lineage of vascular land plants. The group has a broad global distribution ranging from tundra to tropical forests and can make up an important component of temperate northeast US forests. We know very little about the *in situ* ecophysiology of this group and apparently no study has evaluated if lycophytes conform to functional patterns expected by the leaf economics spectrum hypothesis. To determine factors influencing photosynthetic capacity (*A*_max_), we analysed several physiological traits related to photosynthesis to include stomatal, nutrient, vascular traits, and patterns of biomass distribution in four coexisting temperate lycophyte species: *Lycopodium clavatum*, *Spinulum annotinum*, *Diphasiastrum digitatum* and *Dendrolycopodium dendroideum*. We found no difference in maximum photosynthetic rates across species, yet wide variation in other traits. We also found that *A*_max_ was not related to leaf nitrogen concentration and is more tied to stomatal conductance, suggestive of a fundamentally different sets of constraints on photosynthesis in these lycophyte taxa compared with ferns and seed plants. These findings complement the hydropassive model of stomatal control in lycophytes and may reflect canalization of function in this group. Our data also demonstrate functional ecological similarities: *De. dendroideum* and *D. digitatum* are species that have substantial belowground biomass investment and are consistently more similar to each other across multiple traits than either is to the more surficial *S. annotinum* and *L. clavatum*. Such differences may partition environments in ways that allow for the close coexistence of these species.

## Abstract (precis)

Modern clubmosses are a vestige of their gargantuan carboniferous ancestors that dominated the paleoflora flora for millions of years. Yet little is known of the ecophysiology of these plants. The goal of this paper was to examine four temperate lycophyte taxa that are commonly found in northeast US temperate forests. We evaluated the relationship of several functional parameters and found evidence of functional ecological convergence largely based on growth form. Species with substantial belowground biomass investment are consistently more similar across multiple traits than taxa with rhizomes that are largely aboveground. Such differences may help explain how these taxa partition their environment and frequently grow in dense multispecies stands.

## Introduction

Plant ecophysiology is often presented in terms of functional economics, which describe functional trade-offs with particular suites of traits that result in certain trait correlations (Wright *et al*. 2004, 2007; Chave *et al*. 2009; Wright and Sutton-Grier 2012; Creese *et al*. 2014). A series of studies have suggested that leaf traits including light-saturated net photosynthetic capacity (*A*_max_), dark respiration rate, leaf nitrogen (N), specific leaf area (SLA) and leaf lifespan are governed by the leaf economic spectrum (LES) that limits possible trait combinations (Reich *et al*. 1997; Wright *et al*. 2004). For example, *A*_max_ and leaf N concentration (leaf %N) often have a strong positive correlation due to their causal relationship (Wright *et al*. 2004). Plants with high *A*_max_ and high leaf %N typically have a reduced SLA and short lifespan as increased N may attract more herbivores (Wright *et al*. 2004). Such convergence on plant leaf function has been demonstrated across contrasting biomes from deserts to tropical forests and across hundreds of species from herbs to evergreen trees and ferns to flowering plants (Reich *et al*. 1997; Ackerly and Reich 1999; Wright *et al*. 2007; Zhang *et al*. 2015).

The LES hypothesis is widely supported across diverse biomes and phylogenetic groups, yet such correlations are not always the rule within habitats where physiology may be more sensitive to local micro-environmental conditions. For example, photosynthesis can be limited by water relations, especially through stomatal regulation (Brodribb 2009; Brodribb *et al*. 2009; McAdam and Brodribb 2012*b*). Stomatal conductance (*g*_s_) drives transpiration rate, regulates intracellular partial pressure of CO_2_ (*C*_i_), photosynthetic rate and water use efficiency (WUE). Stomata balance these trade-offs by maximizing carbon (C) fixation while minimizing water loss. Stomatal response tends to be under fine-scale control, resulting in outcomes that may differ from those predicted by the LES.

We have a robust understanding of seed plant ecophysiology, yet our knowledge of lycophytes is poor and we know of very few articles that examine *in situ* ecophysiology in general. Such poor understanding is surprising given the size of the group, over 1300 species (Ambrose 2013), and the evolutionary position of the clade: lycophytes are sister to vascular plants. The limited data we have suggest that the unique leaves of lycophytes (microphylls) can have long lifespans (to several years, e.g. Callaghan 1980) with unexpectedly high maximum photosynthetic rates: as high as 6.2 µmol CO_2_ m^−2^s^−1^ in some tropical species (Brodribb *et al*. 2007). The past decade has seen numerous articles evaluating stomatal function in ferns and lycophytes (Franks and Farquhar 2007; Brodribb *et al*. 2009; Brodribb and McAdam 2011; Ruszala *et al*. 2011; McAdam and Brodribb 2012*b*, 2013). Some of these studies suggest that lycophyte stomata function in ways different than seed plants. Most importantly is that some members of the group fail to respond to the hormone abscisic acid (ABA) as other seed plants do (Brodribb and McAdam 2011; McAdam and Brodribb 2012*a*, *b*, 2013), and stomatal function may be more driven by environmental cues such as leaf water status and/or light (Brodribb *et al*. 2009; Chater *et al*. 2013; Kollist *et al*. 2014). If lycophyte stomatal control is inherently different than that in angiosperms and gymnosperms, it is possible that lycophytes will be subject to the different functional trait correlations relative to those seen in other groups.

In addition to stomatal regulation, plant hydraulics also affects whole-plant physiology (Sack and Holbrook 2006; Sack *et al*. 2015 and references therein). Several studies have shown that hydraulic conductivity is directly tied to photosynthesis (Brodribb and Holbrook 2004; Santiago *et al*. 2004; Sack and Holbrook 2006; Watkins *et al*. 2010; Pittermann *et al*. 2013; Brodersen *et al*. 2014). Xylem anatomy, especially conduit diameter and length, exerts significant control over water flow (Pittermann and Sperry 2003; Pittermann *et al*. 2011, 2013). In general, conductivity increases with increasing tracheid diameter, length, and quantity and pit area aperture (Pittermann and Sperry 2003; Sperry *et al*. 2005, 2006). Little is known about lycophyte hydraulics in general, and the relationship between anatomy and conductivity has not been thoroughly explored.

**Figure 1.**
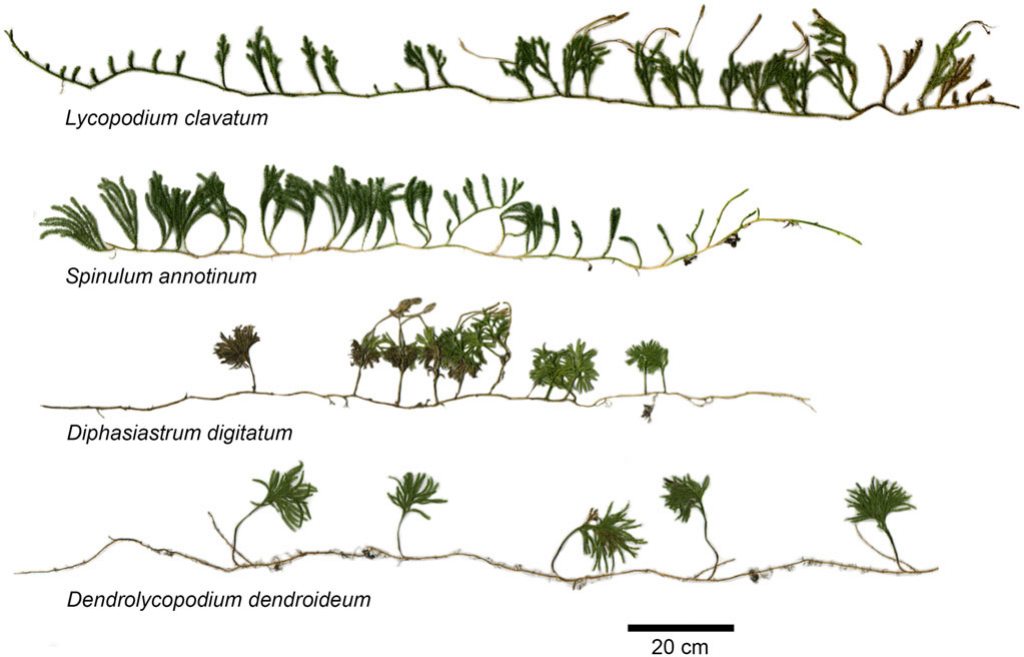
Growth patterns of four lycophyte species. Images produced by scanning whole plants in segments then stitching together in Adobe Photoshop with minimal editing. *Lycopodium clavatum* and *S. annotinum* have photosynthetic microphylls on vertical and horizontal segments, while *De. dendroideum* and *D. digitatum* only have photosynthetic microphylls on vertical segments.

The goal of this article was to examine how four distantly related yet co-occurring lycophyte species behave physiologically in similar habitats. To do this, we evaluate physiological and functional traits in four lycophyte species from the Northeastern USA. Specifically, we evaluate the correlation of functional trait variation related to photosynthesis, nutrient relations, vascular anatomy and growth pattern. The species selected for this study exhibit two growth patterns: *L. clavatum* and *S. annotinum* have both vertical and horizontal photosynthetic shoots and grow primarily aboveground, while *D. digitatum* and *Dendrolycopodium dendroideum* only have vertical photosynthetic shoots aboveground and with more developed horizontal shoots and relatively more dense roots belowground (Fig. 1). We predicted that these growth patterns facilitate niche partitioning that could allow for the co-occurrence of these taxa.

## Methods

The study was conducted in a 300-acre natural wooded area on the Colgate University campus in Hamilton, New York. The site is a typical secondary growth mixed hardwood forest dominated by maple and beech trees. The site hosts four species of lycophytes: *Lycopodium clavatum*, *Spinulum annotinum*, *Diphasiastrum digitatum* and *De. dendroideum*, which are all common and abundant throughout the site. These represent four of the nine major North American genera of lycophytes (Benca 2014). Field data were recorded in September 2014.

### Light response data

Light response data were generated using a portable infrared gas analyser, Li-Cor model LI-6400XT Photosynthesis System (LI-COR Biosciences Inc., Lincoln, NE, USA), during daylight hours between 11:00 and 18:00. We tested 10 widely spaced, vertical ramets per species. We ran an abbreviated light response programme consisting of 6-min intervals at each of the following levels of photosynthetically active radiation (PAR): 800, 50, 25, 15, 10, 5 and 0 μmol m^−2^ s^−1^. The reading at 800 μmol photons m^−2^ s^−1^ served as a measure of light-saturated net *A*_max_ for all species, while the slope of points 50–0 μmol m^−2^ s^−1^ was used to estimate quantum yield. Measures of *g*_s_ were recorded at 800 μmol m^−2^ s^−1^. The sections used for light response data were collected and refrigerated until they were used for area calculations by scanning stems using the software ImageJ (http://imagej.nih.gov/ij/).

### Stomatal density

All four species produce long creeping horizontal rhizomes that vary in soil depth across the species. On the extremes for example, in *L. clavatum*, these rhizomes are almost always above the soil surface yet send adventious roots into the soil. In *De. dendroideum*, the rhizomes are always rooted in the soil. For this reason, we choose to examine rhizomes in all species and refer to this tissue as horizontal segments. Ten samples of each species (including vertical and horizontal segments, Fig. 1) were collected for comparative anatomy. These were fixed in FAA [2 formalin: 10 ethanol (95%): 1 glacial acetic acid: 7 distilled water] and analysed for stomatal density by counting the number of stomata per unit area at three different positions per microphyll. We evaluated 10 microphylls each for vertical and horizontal segment per species when possible. Horizontal segments stomatal data were only acquired for *L. clavatum* and *S. annotinum* because *De. dendroideum* and *D. digitatum* do not have photosynthetic microphylls on horizontal segments.

### Biomass distribution

Five complete plants of each species were collected for vertical and horizontal biomass comparisons. A single clone consisted of all horizontal and vertical segments, strobili and roots from the most apical segment of the clone to the oldest segments possible to extract (Callaghan 1980). Beyond this point, the clone is impossible to extract because it is too decomposed. Vertical and horizontal segments were separated, oven-dried at 70 °C for 4 days and weighed.

### Nutrient analysis

Vertical tissues collected for biomass measurements and the samples that were used to measure photosynthesis were ground into a fine powder using a SPEX SamplePrep 5100 Mixer/Mill (SPEX SamplePrep, Metuchen, NJ, USA). Next, 3.2–3.7 mg quantities of each sample were rolled into tin capsules for elemental analysis. Samples were processed using a Costech Elemental Analyser (Costech Analytical Technologies, Inc., Valencia, CA, USA) for %C and %N.

### Vascular anatomy

To assess vascular anatomy, 10 samples of each species (5 horizontal and 5 vertical) were heated for ∼3 weeks at 90 °C in a maceration solution (1 : 4 : 5, 30 % hydrogen peroxide : deionized water : acetic acid). After samples were translucent and soft tissue was mostly dissolved, samples were stored in vials of 70 % EtOH. Tracheids were isolated, stained with safranin and observed under ×40 magnification. For each sample, 10 randomly selected tracheids that were undamaged and identifiable along their entire lengths were chosen and photographed using PAX-It Imaging Software (MIS Inc., Villa Park, IL, USA). When necessary, images were stitched together using Adobe Photoshop (Adobe, San Jose, CA, USA). Tracheid lengths were subsequently measured using Image-J. Total vascular area and individual tracheid areas were measured by making hand cross-sections of five horizontal and five vertical shoot samples per species. Images of cross-sections were captured using PAX-It Imaging Software. Using the same programme, the total vascular area and the areas of five individual tracheids were measured.

### Statistical analysis

One-way analyses of variance were performed to compare *A*_max_, *g*_s_, stomatal density, WUE and %N in vertical segments among species, stomatal density and tracheid lengths between vertical and horizontal segments among species and horizontal : vertical biomass ratio among species. *Post hoc* Tukey tests were used to determine homogeneous subsets. Independent samples *t*-tests were performed to compare stomatal density and tracheid length between vertical and horizontal shoots within species. Bivariate regressions were used to evaluate the relationships between *A*_max_ and *g*_s_, %N and *g*_s_ × *C*_i_. All analyses were performed using JMP ver. 10 statistical software (SAS Institute Inc., Cary, NC, USA) and figures were produced in SigmaPlot ver. 13 (Systat Software Inc., San Jose, CA, USA).

## Results

**Figure 2.**
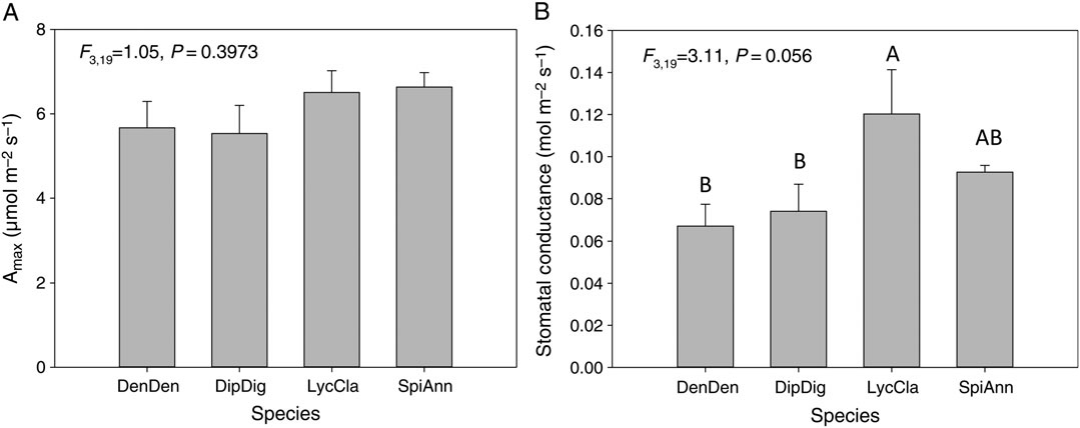
(A) Maximum area-based photosynthetic rate (*A*_max_) and (B) *g*_s_ of four lycophyte species measured after 6 min at 800 μmol photons m^−2^ s^−1^, *n* = 5 per species. Error bars are SE. Species abbreviations are as follows: DenDen, *De. dendroideum*; DipDig, *D. digitatum*; LycCla, *L. clavatum*; and SpiAnn, *S. annotinum*.

**Figure 3.**
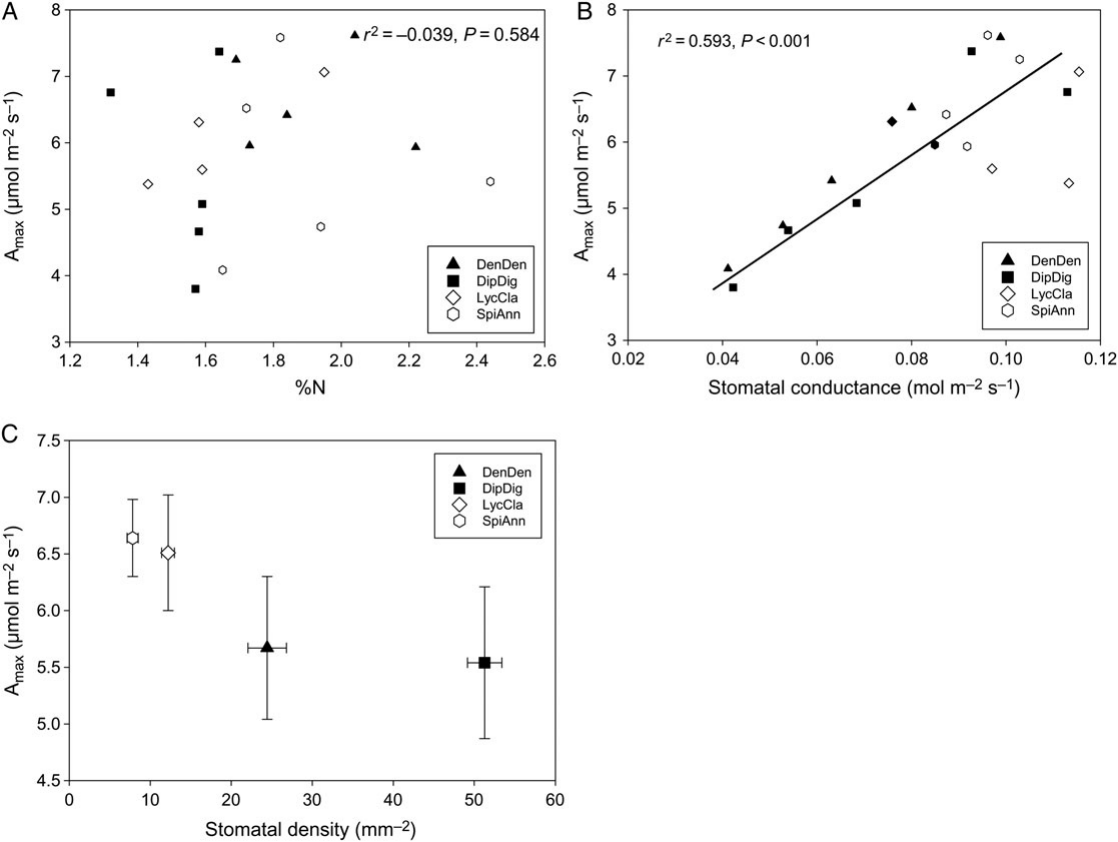
Maximum area-based photosynthetic rate (*A*_max_) at 800 μmol photons m^−2^ s^−1^ versus (A) %N of vertical segments, (B) *g*_s_ measured at 800 μmol photons m^−2^ s^−1^ and (C) stomatal density of vertical segments in four lycophyte species. Species data are combined in (A) and (B). Error bars in (C) are SE.

**Figure 4.**
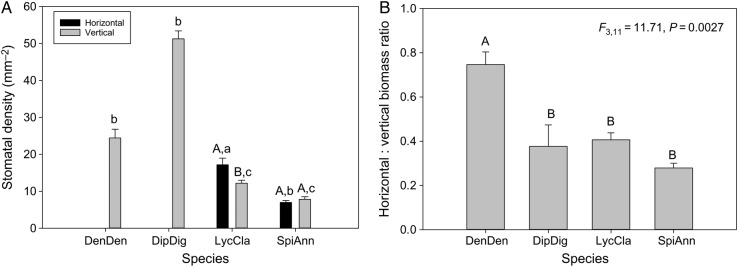
(A) Stomatal density and (B) biomass allocation in four lycophyte species. Error bars are SE. Uppercase letters designate interspecies homogenous subsets, while lowercase letters designate intraspecies homogeneous subsets.

**Figure 5.**
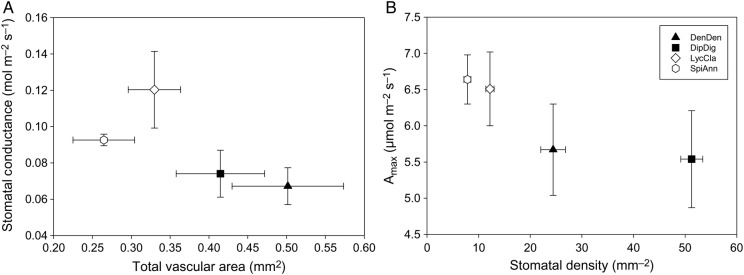
Relationship between total vascular area of vertical segments and (A) *g*_s_ and (B) *A*_max_. Error bars are SE.

Across several variables, *D. digitatum* and *De. dendroideum* grouped together relative to *L. clavatum* and *S. annotinum*. The four species in this study exhibited essentially two growth patterns: surficial and deeply rooted horizontal segments (Fig. 1). Despite these qualitative differences in photosynthetic investment, these species exhibit no statistically significant differences in *A*_max_ across species (*F*_3,19_ = 1.05, *P* = 0.3973, Fig. 2A). Nor do any differences exist in quantum yield among species (*F*_3,19_ = 1.883, *P* = 0.173, data not shown). Differences in *g*_s_ were marginally significant among species (*F*_3,19_ = 3.11, *P* = 0.056, Fig. 2B), with *L. clavatum* and to a lesser extent *S. annotinum* exhibiting higher *g*_s_. Stem %N did differ significantly among species (*F*_3,19_ = 3.41, *P* = 0.0431, data not shown), but no relationship was found between *A*_max_ and %N in vertical segments in species individually or when pooled (*r*^2^ = −0.039, *P* = 0.584, Fig. 3A). However, a strong correlation was found between *A*_max_ and *g*_s_ for *D. digitatum* (*r*^2^ = 0.843, *P* = 0.028) and *De. dendroideum* (*r*^2^ = 0.991, *P* < 0.0001) with a weaker correlation in *L. clavatum* (*r*^2^ = 0.605, *P* = 0.121) and *S. annotinum* (*r*^2^ = 0.581, *P* = 0.134); the correlation was significant when all species were combined together (*r*^2^ = 0.593, *P* < 0.0001 Fig. 3B). There was no significant relationship between *A*_max_ and stomatal density (Fig. 3C). Although *D. digitatum* and *De. dendroideum* have higher stomatal densities on vertical segments than *L. clavatum* and *S. annotinum* (*F*_3,39_ = 135, *P* < 0.0001, Fig. 4A), the species exhibit differences in horizontal : vertical biomass distribution, with *De. dendroideum* having significantly more horizontal biomass relative to vertical biomass compared with the other three species, which all have significantly more vertical than horizontal biomass (*F*_3,11_ = 11.71, *P* = 0.0027, Fig. 4B). Patterns of tracheid lengths are consistent among species, with horizontal segments in all species having mean horizontal tracheid lengths roughly two times that of vertical segments (4.07 and 2.1 mm, respectively; all *P*-values <0.0001). Horizontal tracheid lengths are significantly different among species (*F*_3,199_ = 3.27, *P* = 0.022), but vertical tracheid lengths are not (*F*_3,199_ = 0.344, *P* = 0.837). Patterns in total vascular area varied among species. Total horizontal vascular area did not differ among species (*F*_3,17_ = 2.189, *P* = 0.135), while differences did exist in total vertical vascular area (*F*_3,19_ = 4.00, *P* = 0.0265). *Diphasiastrum digitatum*, *L. clavatum* and *S. annotinum* had significantly larger total horizontal vascular area than in vertical segments (all *P*-values <0.05), while total vascular area was the same in horizontal and vertical segments in *De. dendroideum* (*F*_3,9_ = 0.787, *P* = 0.401). Total vertical vascular area was not correlated with *g*_s_ (Fig. 5A) or *A*_max_ (Fig. 5B).

## Discussion

Our understanding of basic ecophysiology of lycophytes is limited, and our data are the first of their kind to analyse physiological ecology of temperate lycophyte taxa in a field setting. The four taxa in this study grow in similar habitats, growing closely together and often intertwined. This provided us with a unique opportunity to evaluate physiological differences in what was essentially a common garden experiment. We failed to find significant differences in maximum photosynthetic rates of the four species in this study (Fig. 2A), but a number of analyses revealed patterns where *De. dendroideum* and *D. digitatum* were similar to each other relative to the other pair, *S. annotinum* and *L. clavatum* (Figs 3C, 4A and 5). We found surprising variation in maximum photosynthetic rates with the four species combined averaging 6.1 μmol m^−2^ s^−1^ (low in *D. digitatum* 3.801 μmol m^−2^ s^−1^ to high in *L. clavatum* 8.191 μmol m^−2^ s^−1^). The mean is two times as large as previously published measures for *S. annotinum* and *L. clavatum*, which Brodribb *et al*. (2007) found to be ∼3 μmol m^−2^ s^−1^. Such discrepancies are difficult to explain especially in light of the extreme paucity of gas exchange data on lycophytes. It is possible that the two studies simply represent variation within these species that occur across different sites.

We did not find a significant correlation between area-based *A*_max_ measurements and %N in vertical tissues when species were analysed either individually or as a composite. Reich *et al*. (1997), and several other studies (e.g. Hikosaka and Hirose 2001; Hikosaka 2004; Takashima *et al*. 2004; Wright *et al*. 2004, 2005; Muller *et al*. 2011) have reported a tight relationship between net photosynthesis and leaf %N for many species across a diverse array of habitats when these traits are expressed on a mass basis. The hypothesis that plant functional traits are related in a predictable manner has been called the global leaf economics spectrum. This also holds, yet such relationships are weaker when area-based measurements are incorporated (e.g. Evans 1989). That we failed to find a correlation between area-based *A*_max_ and %N in these lycophyte species is perhaps not surprising given our relatively small sample size of species and limited community sampling. However, Wright and Sutton-Grier (2012) evaluated trait trade-offs in a small community of co-occurring species and their results provided only modest support for previously reported trait linkages. Clearly, more work needs to be completed on lycophytes and future studies should incorporate mass-based measurements to better evaluate how this group conforms to trait correlations expected under the global leaf economics spectrum.

It is possible that lycophytes deviate from such expectations given potentially unusual stomatal function relative to other plants (Brodribb and McAdam 2011; McAdam and Brodribb 2012*a*, 2013; Chater *et al*. 2013; Chater and Gray 2015). Although we did not measure hydraulics directly, our analyses of anatomical variables frequently related to hydraulic conductance were unrelated to photosynthesis and *g*_s_ (Fig. 5). We did find that *g*_s_ was correlated with *A*_max_ across all species, but this was, in part, driven by strong correlations of *De. dendroideum* and *D. digitatum* (Fig. 3B). The relation between *g*_s_ and *A*_max_ does not appear to be related to stomatal density (Fig. 3C). Stomatal density did vary significantly among the four species in the vertical portions and this seems to be driven by photosynthetic tissue allocation, as *S. annotinum* and *L. clavatum* have stomata on both horizontal and vertical segments. Stomatal densities between vertical and horizontal segments were similar between these two species, albiet slightly higher in *L. clavatum* (Fig. 4B). Unfortunately, we did not measure *g*_s_ in these horizontal segments. We did find that across all four species, %N was always higher in the vertical segments (data not shown). It would be quite interesting for future work to evaluate how these segments function and whether or not they contribute to the C of individual plants.

Many of our analyses produce patterns where *De. dendroideum* and *D. digitatum* were more similar to each other relative to *S. annotinum* and *L. clavatum*. These groupings may reflect the unique ecology of these species. One particularly interesting aspect of the biology of these species is that all four can be found growing intermixed in dense populations. Each species requires a unique niche and what we see aboveground may not reflect what happens belowground. *Dendrolycopodium dendroideum* and *D. digitatum* invest more biomass in belowground structures that are non-photosynthetic, whereas *S. annotinum* and *L. clavatum* produce photosynthetic rhizomes that run along the surface of the soil. Such differences may partition belowground environments in ways that allow for the close coexistence of these species. Similar patterns of belowground niche partitioning have been reported for other taxa (e.g. Jones *et al*. 2011; Paz *et al*. 2015) and resource partitioning in general has been used to explain species densities and distributions in several other studies (e.g. Tilman *et al*. 2001; Kamiyama *et al*. 2014). We know of no other study that has attempted to evaluate partitioning in lycophytes, but given close physical occurrence of these four species together in many northeast temperate forests in the USA, this is an excellent area for future investigation.

The poor correlation between stomatal density and conductivity suggests that intrinsic stomatal behaviour could be driving photosynthetic rates rather than stomatal number. A series of articles demonstrate that lycophytes regulate *g*_s_ differently than seed plants (Brodribb 2009; Brodribb *et al*. 2009; Brodribb and McAdam 2011; McAdam and Brodribb 2012*a*). Whereas seed plant stomata are highly responsive to the hormone ABA, several studies suggest that lycophyte stomata have passive stomatal control based on leaf water potential (Brodribb and McAdam 2011; McAdam and Brodribb 2012*a*). Studies on other lycophyte taxa have demonstrated low stomatal responsiveness to ambient CO_2_ concentration and light intensity (Brodribb *et al*. 2009; McAdam and Brodribb 2012*a*) and insensitivity to ABA (Brodribb and McAdam 2011; McAdam and Brodribb 2012*b*). Our understanding of lycophyte stomatal behaviour is in a state of flux, and more work needs to be done to understand how stomatal function in this group is related to photosynthesis and ecology.

## Sources of Funding

Our work was funded through a grant from the Colgate University Research Council and The Picker Interdisciplinary Research Institute at Colgate University.

## Contributions by the Authors

All authors contributed to the data collection, analysis and writing. The author for correspondence was also responsible for gathering funding for the work.

## Conflict of Interest Statement

None declared.
